# Functional screening for triclosan resistance in a wastewater metagenome and isolates of *Escherichia coli* and *Enterococcus* spp. from a large Canadian healthcare region

**DOI:** 10.1371/journal.pone.0211144

**Published:** 2019-01-24

**Authors:** Andrew Cameron, Ruth Barbieri, Ron Read, Deirdre Church, Emelia H. Adator, Rahat Zaheer, Tim A. McAllister

**Affiliations:** 1 Faculty of Veterinary Medicine, University of Calgary, Calgary, AB, Canada; 2 Lethbridge Research and Development Centre, Lethbridge, AB, Canada; 3 Faculty of Medicine, University of Calgary, Calgary, AB, Canada; 4 Calgary Laboratory Services, Calgary, AB, Canada; 5 Department of Food and Human Nutritional Sciences, University of Manitoba, Winnipeg, MB, Canada; Aligarh Muslim University, INDIA

## Abstract

The biocide triclosan is in many consumer products and is a frequent contaminant of wastewater (WW) such that there is concern that triclosan promotes resistance to important antibiotics. This study identified functional mechanisms of triclosan resistance (TCS^R^) in WW metagenomes, and assessed the frequency of TCS^R^ in WW-derived and clinical isolates of *Escherichia coli* and *Enterococcus* spp. Metagenomic DNA extracted from WW was used to profile the microbiome and construct large-insert cosmid libraries, which were screened for TCS^R^. Resistant cosmids were sequenced and the TCS^R^ determinant identified by transposon mutagenesis. Wastewater *Enterococcus* spp. (*N* = 94) and *E*. *coli* (*N* = 99) and clinical *Enterococcus* spp. (*N* = 146) and vancomycin-resistant *E*. *faecium* (VRE; *N* = 149) were collected and tested for resistance to triclosan and a comprehensive drug panel. Functional metagenomic screening revealed diverse FabV homologs as major WW TCS^R^ determinants. Resistant clones harboured sequences likely originating from *Aeromonas* spp., a common WW microbe. The triclosan MIC90s for *E*. *coli*, *E*. *faecalis*, and *E*. *faecium* isolates were 0.125, 32, and 32 mg/L, respectively. For *E*. *coli*, there was no correlation between the triclosan MIC and any drug tested. Negative correlations were detected between the triclosan MIC and levofloxacin resistance for *E*. *faecalis*, and between triclosan and vancomycin, teicoplanin, and ampicillin resistance for *E*. *faecium*. Thus, FabV homologs were the major contributor to the WW triclosan resistome and high-level TCS^R^ was not observed in WW or clinical isolates. Elevated triclosan MICs were not positively correlated with antimicrobial resistance to any drug tested.

## Introduction

Triclosan is a synthetic chlorinated bisphenol antimicrobial drug, commonly referred to as a biocide, and is effective against a wide variety of microorganisms, including bacteria, fungi, and apicomplexan parasites [1, 2]. Many clinical and personal hygiene products, such as soaps, sanitizers, and toothpaste include triclosan at concentrations up to 0.3% (300 g/L or ~1 M). Triclosan is also found in human urine [3], milk [4], wastewater (WW) and aquatic systems [5]. The widespread inclusion of triclosan in consumer products is controversial [2], as it is structurally similar to T_3_ and T_4_ thyroid hormones [6, 7] and has the potential to promote antimicrobial resistance (AMR) [5, 8].

Triclosan targets the elongation cycle in bacterial fatty acid biosynthesis by inhibiting enoyl-acyl-carrier-protein reductases (ENRs) [9, 10], disrupting bacterial cell wall function. Currently, there are four well-known ENR isozymes: FabI, FabL, FabV, and FabK, with FabI, FabL, and FabV belonging to the short-chain dehydrogenase/reductase superfamily [10]. Most bacteria encode *fabI*, such that triclosan resistance (TCS^R^) is conferred by mutations or overexpression of FabI, or via efflux pumps [10]. The other isozymes exhibit varied sensitivity to triclosan, with FabV conferring almost complete insensitivity. Of the isozymes, FabV was most recently discovered in *Vibrio cholerae* and has a sequence length that is ~ 60% longer than other reductases [9, 10]. Screening of metagenomic libraries has identified novel TCS^R^ genes such as candidate FabG-like and YX7K-type ENRs, AcrAB efflux pump homologs, and other hypothetical and unknown determinants [11].

Widespread TCS^R^ could pose a public health risk if it promotes antimicrobial resistance (AMR) to clinically important drugs. Acquired TCS^R^ and multidrug-resistance (MDR) in clinical human pathogens is generally infrequent. However, *Staphylococcus aureus* isolates can harbour a second (heterodiploid) TCS^R^ copy of *fabI*, potentially acquired from *Staphylococcus haemolyticus* [12]. Studies have shown that triclosan induces or selects for mutant genes overexpressing MDR efflux pumps. For example, in clinical *Escherichia coli*, deletion mutants of the *acrAB* efflux system are 10-fold more sensitive to triclosan, and overexpression of *acrAB* enhances resistance [13]. Likewise, triclosan selects for *Stenotrophomonas maltophilia* mutants that overexpress the *smeDEF* efflux pump, and have enhanced resistance to tetracycline, chloramphenicol, and ciprofloxacin [14]. Interestingly, FabI (or InhA in *Mycobacterium tuberculosis*) is also the target of isoniazid, an important anti-tubercular agent. Structural and genetic studies on the enzyme indicate that triclosan could select for isoniazid-resistant reductases [15] and triclosan derivatives have been explored as anti-mycobacterial drugs [16].

Recently, functional screening for TCS^R^ in soil metagenomic libraries identified diverse ENR homologues [11]. Additional *in silico* metagenome and genome-wide analyses have also evaluated the distribution of TCS^R^ in pathogenic bacteria derived from potentially triclosan-contaminated environments, and found that long-term and extensive triclosan contamination could lead to the selective emergence of TCS^R^ pathogens [17]. Accurate *in silico* prediction of TCS^R^ depends on current knowledge of TCS^R^ mechanisms. However, compared to AMR, the emergence of resistance to triclosan remains relatively unexplored in many species, including uncultured microbes. In this study, we aimed to explore the WW triclosan resistome utilizing both metagenomic and culture-based approaches. We anticipated that this approach could reveal the diversity and origin of TCS^R^ determinants in environments and microbes potentially exposed to triclosan. Thus, we explored and assessed the frequency of TCS^R^ mechanisms in WW metagenomes by constructing, screening, and sequencing large-insert cosmid libraries. We also profiled the WW microbiome, and collected both sentinel WW *E*. *coli* and *Enterococcus* spp.—and comparator clinical *Enterococcus* spp.—to correlate susceptibility to triclosan against a panel of relevant antimicrobials.

## Materials and methods

### Clinical laboratory setting

Calgary Laboratory Services (CLS) is a clinical laboratory that provides diagnostic services to a population of ~1.5 million people. Diagnostic analyses are performed in a single regional laboratory serving both institutionalized and ambulatory patients.

### Sampling and bacterial isolation, strains, plasmids, cosmids, and culturing methods

Between August 2014 and June 2016 bi-monthly samples (*N* = 12) were collected from sewage influent from a municipal WW plant in Calgary, AB (51.0486 N 114.0708 W). *Enterococcus* spp. were isolated using membrane filtration onto modified mEI (BD Difco) agar according to US EPA Method 1600 [18] and mEI containing 8 mg/L erythromycin (*N* = 257). Up to 10 presumptive indoxyl-β-D-glucoside-cleaving blue colonies grown for 48 h at 37°C were selected and streaked onto Bile Esculin Agar (BEA). Colonies were re-streaked on fresh BEA, and cryopreserved at -80°C in Brain Heart Infusion (BHI) broth containing 10% glycerol. For comparison, *Enterococcus* spp. from human clinical samples in the same time period were obtained from CLS. *Enterococcus* spp. were either isolated from urine, superficial wounds, cerebral spinal fluid or blood, whereas vancomycin-resistant Enterococci (VRE) were recovered from nasal or rectal swabs. Clinical isolates were presumptively identified via MALDI-TOF. A suspension of *Enterococcus* sp. (75 μl) in TE pH 7.4 was used for DNA extraction via heat-lysis (5 min 98°C, 5 min centrifugation at 10,000 × g). *Enterococcus* were speciated via PCR and Sanger-based sequencing (Eurofins Genomics) of *groES-EL* [19]. *E*. *coli* and *Enterococcus* spp. were isolated from WW as described previously[19].

For routine culturing, *Enterococcus* spp. and *E*. *coli* were grown on BHI agar. Where appropriate, *E*. *coli* strains harbouring pCC2FOS, triclosan resistance determinants, or transposon insertions were selected for and maintained with either LB agar or broth supplemented with chloramphenicol (15 mg/L, MilliporeSigma), triclosan (5 mg/L; MilliporeSigma), or tetracycline (10 mg/L; MilliporeSigma), respectively. Triclosan was constituted as a 10 g/L stock in dimethyl sulfoxide (DMSO).

### Metagenomic DNA cosmid library construction and screening

Wastewater samples were centrifuged (10 min, 8000 × g) and metagenomic DNA was extracted from 200 mg pellets obtained from ~120 mL of centrifuged influent, corresponding to samples obtained in December 2014 (CST062), June 2015 (CST096), and August 2015 (CST117). DNA was extracted following repeated bead-beating, chemical lysis, and column-based purification (QIAamp DNA stool kit, Qiagen) [20]. Large-insert expression libraries were constructed for each sample according to the manufacturer’s recommendations for the pCC2FOS CopyControl Fosmid Library Production Kit (Lucigen) [21]. To screen for triclosan resistance, 5 mL of Mueller-Hinton (MH) broth was inoculated (initial OD_600_ 0.5) with the pooled cosmid strain libraries, and grown for ~2 h at 37°C. Resulting cultures were standardized to 0.1 OD_600_ from which a 10-fold dilution series was plated for enumeration on MH, and MH supplemented with triclosan (5 mg/L, MilliporeSigma) or chloramphenicol (15 mg/L). Empty vector *E*. *coli* EPI300 and control libraries (constructed separately from DNA harvested from *E*. *coli* ATCC 25922 or *E*. *faecalis* ATCC 51299) were also screened. Concentrations of triclosan for screening were selected based on previous literature [10, 22]. Cosmids were extracted from 48 TCS^R^ colonies (BioBasic EZ-10 Spin Column Plasmid DNA Miniprep kit), and profiled for uniqueness via restriction enzyme digestion with *Eco*RI (New England Biolabs). Those with unique *Eco*RI restriction patterns were electroporated into *E*. *coli* DH5α for TCS^R^ confirmation.

### Sequencing, bioinformatics, and cosmid transposon insertion mapping

Fifteen cosmids were submitted to Génome Québec Innovation Centre for Illumina MiSeq PE250 shotgun sequencing. Trimmomatic 0.36 [23] was used to trim paired reads and the Illumina adaptor (BioO) with the following criteria (phred33, LEADING:3 TRAILING:3 SLIDINGWINDOW:4:15 MINLEN:36), resulting in read retention of >98%. Reads aligning with the genome of *E*.*coli* K12 (NCBI RefSeq NC_000913.3) were eliminated. Default settings in SPAdes 3.10.1 [24] and PROKKA [25] were used for assembly and annotation, respectively. For all cosmids, assembly resulted in a single contig containing the pCC2FOS sequence (GenBank: EU140752.1), which was subtracted in Geneious 8.19. Additional annotation and analysis was performed with RAST [26].

To identify TCS^R^ cosmids, *in vitro* transposition (tetracycline-resistant EZ-Tn5 Insertion Kit, Lucigen) was used to create transposon mutant libraries for 5 cosmids selected based on lowest sequence identity. Cosmid DNA was mutagenized and transformed into electrocompetent *E*. *coli* DH5α. Following recovery in SOC broth, electroporated cells were plated on LB with tetracycline (10 mg/L) and grown for 24–48 h at 37°C. Recovered transformants were streaked in parallel onto fresh LB with tetracycline or triclosan. Cosmids were extracted from transformants that failed to grow on triclosan and the transposon was mapped using oligonucleotides (TET-1 FP-1 and RP-1) via Sanger sequencing (Eurofins Genomics). Up to 12 insertions were mapped to each cosmid in Geneious 8.1.9. Following identification of the resistance gene, the syntenous ORF was identified in the remaining TCS^R^ cosmids.

For metagenomic analyses, DNA samples from the cosmid libraries were sequenced via Illumina HiSeq Rapid PE250, using SAGE cassettes for DNA gel extraction and NEB Ultra II library preparation. Bioinformatic analyses were performed in Galaxy [27] at the National Microbiology Laboratory, Public Health Agency of Canada. Adapters and low quality reads were removed with Trimmomatic 0.36 [23] using phred15, LEADING:3 TRAILING:3 SLIDINGWINDOW:4:15 MINLEN:36 as parameters. Taxonomic reads were classified with Kraken [28] as previously described [20]. For *fabV* read mapping, BWA-MEM 0.7.17.1 was run with trimmed paired reads using default settings on simple Illumina mode [29], with terminal reads manually filtered. The top BLAST alignment was used to assign species to each filtered read.

### Triclosan and antimicrobial susceptibility assays

Triclosan susceptibility was assessed using the microtiter broth dilution method for MIC determination [30]. Triclosan concentrations tested for *Enterococcus* spp. were 0.125–128 mg/L and 0.0078–8 mg/L. for *E*.*coli*. *E*. *coli* ATCC 25922, *E*. *faecalis* ATCC 29212, and *E*. *coli* DH5α harbouring the high-level triclosan-resistance plasmid pF2 [22] were included as controls. Standard visual inspection to determine MIC was not used because of the insolubility of triclosan in MH broth. Rather, MICs were determined by measuring OD_600_ in a plate reader (BioTek Synergy HTX), and defined as the triclosan concentration which inhibited growth in a manner comparable to a control dilution series. For all other drugs, disk susceptibility tests were conducted according to CLSI guidelines (CLSI document M02-A12 and CLSI supplement M100S) [31, 32]. Transformed *E*. *coli* DH5α and EPI300 were tested for resistance to tetracycline, clindamycin, nalidixic acid, enrofloxacin, ceftriaxone, rifampin, polymixin B, nitrofurantoin, sulfamethoxazole, sulfisoxazole, kanamycin, gentamicin, and spectinomycin. *E*. *coli* isolated from WW were screened for ampicillin, ceftazidime, amoxicillin/clavulanate, ceftiofur (Oxoid), streptomycin, sulfisoxazole, neomycin, florfenicol (Oxoid), trimethoprim/sulfamethoxazole, and oxytetracycline resistance. Antimicrobials tested for *Enterococcus* spp. included vancomycin, doxycycline, tigecycline (Oxoid), levofloxacin, linezolid (Oxoid), erythromycin, streptomycin HLAR, gentamicin HLAR, ampicillin, nitrofurantoin, quinupristin/dalfopristin, and teicoplanin (Oxoid). Zones of inhibition were assessed with the BioMic V3 imaging system (Giles Scientific) and classified as sensitive or resistant based on CLSI criteria, except for tigecycline and neomycin which used EUCAST criteria (www.eucast.org).

### Statistical analyses

Statistical tests were performed in Sigmaplot 13.0 (Systat Software Inc.), with error bars indicating standard error of the mean. For triclosan MIC and antimicrobial resistance correlations, Spearman’s Rank-Order was used. For pairwise sequence comparisons, *fabV* was translated (bacterial transl_table 11), aligned with default MUSCLE parameters in Geneious 8.1.9, and maximum likelihood-based phylogenetic trees were constructed with PhyML 3.0 Smart Model Selection [33].

### Nucleotide sequence accession numbers

Illumina sequence data were deposited in the NCBI Short Read Archive under BioProject ID PRJNA482680. Cosmid insert sequences were deposited in GenBank under accession numbers MH687380-MH687394.

## Results

### Construction of large-insert metagenomic cosmid libraries

The number of clones recovered from the 3 pooled large-insert DNA cosmid WW libraries (CST062, CST096, and CST117) were ~38K, ~41K, and ~37K, respectively. Assuming a consistent insert size of ~40 kb, this represents ~4.7 Gbp encoded in ~118,000 clones. Random selection, cosmid extraction, and *Eco*RI digestion of 72 representative clones indicated a high insert diversity. The two genomic control libraries constructed with DNA from *E*. *coli* and *E*. *faecalis* were comprised of 22.5K and 20K clones respectively, and with genome sizes of ~5.2 and ~3.0 Mb, it was estimated that 596 and 343 clones (each 35 kb) provided 99.9% genomic coverage [34].

### Triclosan resistance gene identification

**Fig 1:**
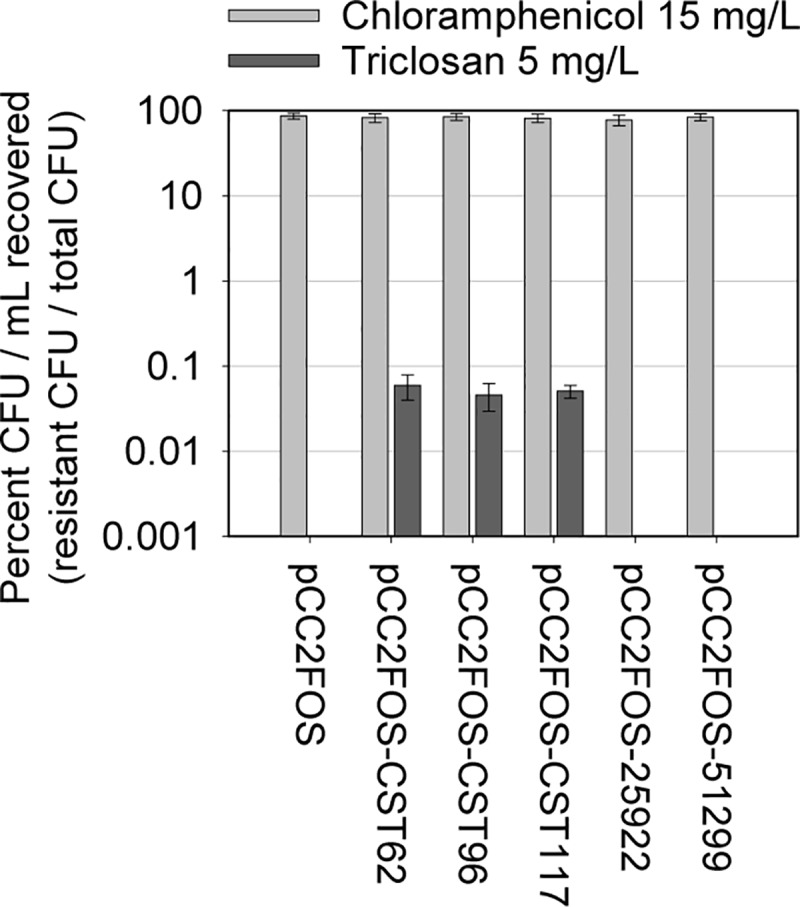
Frequency of functional triclosan resistance in waste water metagenomic libraries. Pools of cosmid strains harbouring WW metagenomic DNA (or controls) were standardized by OD_600nm_ and a 10-fold dilution series was plated for CFU enumeration on LB agar (total CFU) or on LB agar supplemented with chloramphenicol (light grey; to enumerate bacteria harbouring the cosmid vector) or triclosan (dark grey; to select and enumerate triclosan-resistant colonies). Bar graph (logarithmic scale) shows resistant CFU recovered as a percent of total CFU. Mean results with SEM from three independent experiments are shown.

**Fig 2:**
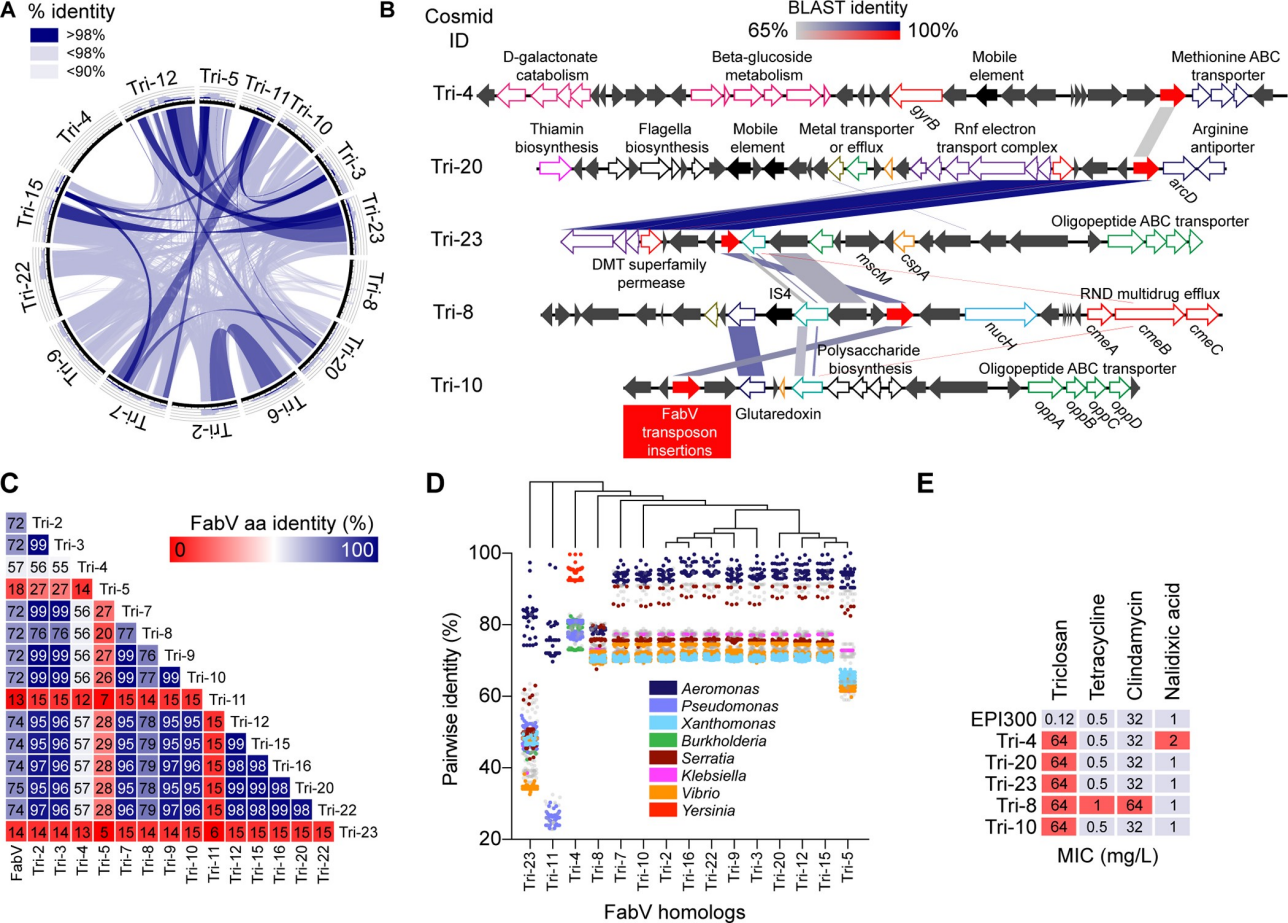
FabV homologs contribute to the waste water triclosan resistome. **(A)** Circular plot[35] indicating synteny (% sequence identity) between metagenomic DNA sequences recovered from triclosan-resistant cosmids. Histograms indicate frequency of shared alignment. **(B)** Identification of triclosan-resistant *fabV* homologs (red arrows) in representative cosmid metagenomic DNA sequences via *in vitro* transposon mutagenesis. Linear BLAST comparison with maximum identity shown as blue or red (inverted). Predicted ORFs are depicted as arrows, with coloured outlines indicating functional categories shown in Fig 2E. **(C)** FabV amino acid (aa) identity matrix comparing the predicted sequence of FabV homologs identified in this study and the *Vibrio cholerae* FabV prototype. Numbers indicate % aa identity**. (D)** Dot plot showing pairwise nucleotide identity of the top 500 *nr* BLAST hits for each FabV homolog, with notable species coloured as indicated. Data arranged according to maximum likelihood-based phylogenetic tree. (**E)** Minimum inhibitory concentrations (MICs) of select antimicrobials determined for representative cosmid strains.

No TCS^R^ colonies were detected in any of the control libraries, with TCS^R^ colonies in WW libraries comprising 0.052 ± 0.004% of total clones (Fig 1). A total of 48 TCS^R^ colonies were subcultured for isolation and extraction of cosmid DNA, which was digested with *Eco*RI to profile and eliminate identical clones. Fifteen unique cosmids were sequenced resulting in ~165,000 reads for each cosmid (insert length min/median/max/: 18,230/31,353/37,180 bp; maximum depth of coverage: ~460X). Despite unique restriction profiles, BLAST comparisons indicated partial synteny between some cosmids (Fig 2A), and excepting Tri-4, when compared to the the *nr* database all inserts appeared to originate from the chromosomal genomes of *Aeromonas hydrophila* or *Aeromonas media*. The Tri-4 cosmid had 99.6% pairwise identity (to 47.8% of the cosmid sequence) to *Yersinia intermedia*. Gene annotation did not conclusively identify TCS^R^ determinants, while several cosmids carried putative efflux pumps. Thus, to functionally identify putative TCS^R^ genes, the five cosmids exhibiting the least synteny were selected for transposon mutagenesis (Fig 2B). Mapping of triclosan-sensitive transposon mutants identified multiple insertions in one gene (or its promoter) in each cosmid annotated as “conserved hypothetical protein” or “putative reductase”. The corresponding genes in non-mutagenized cosmids were identified by pairwise sequence similarity. BLAST alignment, and conserved domain homology, suggested that the genes identified encode ENRs, specifically FabV homologs. These homologs had 13% - 75% amino acid (aa) identity to a prototype FabV (UniProtKB accession: Q9KRA3) (Fig 2C) and high nucleotide identity (>80%) to putative FabV reductases in *Aeromonas* spp. (Fig 2D). They also shared high identity (~90%) with genes in some *Serratia* spp. The Tri-4-encoded FabV had high aa identity (92–99%) with reductases in multiple *Yersinia* spp.; including *Y*. *enterocolitica* and Y. *pestis*.

### Triclosan resistance gene context and taxonomic placement

Several *fabV* were encoded near homologs of *zupT* heavy metal transporters or putative heavy metal efflux pumps, which may be involved in heavy metal tolerance. However, microtiter assays using MH broth supplemented with 2-fold dilutions of ZnCl_2_, ZnSO_4_, Na_2_MoO_4_, CuSO_4_, MnSO_4_, or FeSO_4,_ did not reveal any MIC differences among cosmid strains. Contrarily, cosmid strains that harboured *zupT* were found to exhibit an extended lag phase in the presence of 4 mM ZnCl_2_. Some transposases and genes associated with phage were detected near *fabV*, but otherwise no cosmid insert appeared to harbour integrons or horizontal gene transfer mechanisms. Overall, *fabV* loci appeared to encode a variety of housekeeping functions, such as for carbohydrate metabolism, electron transfer, or flagella biosynthesis. Compared to the RAST subsystem distribution of the genome of *A*. *hydrophila* subsp. *hydrophila* ATCC 7966T [36], glutaredoxin and other glutathione homeostasis genes were over-represented in cosmid sequences (comprising 16% of annotated genes, up from 6%).

Only modest, 2-fold MIC increases were seen for cosmids Tri-4 (MIC increasing from 1 to 2 mg/L for nalidixic acid) and Tri-8 (MIC increasing from 0.5 and 32 mg/L to 1 and 64 mg/L for tetracycline and clindamycin, respectively) (Fig 2E). Tri-4 harbours a copy of *gyrB*, and Tri-8 harbours a putative three gene RND multidrug efflux pump, annotated as *cmeABC*. This version of the efflux pump shared up to 90% aa identity with the *cmeB* gene in *S*. *maltophilia* K279a, yet the cognate *cmeA* and *cmeC* shared only 62% and 54% identity, respectively. The *cmeABC*-encoded efflux pump was not involved in TCS^R^, as deletion of the Tri-8 FabV homolog abolished the TCS^R^ phenotype.

### Wastewater community profile

**Fig 3:**
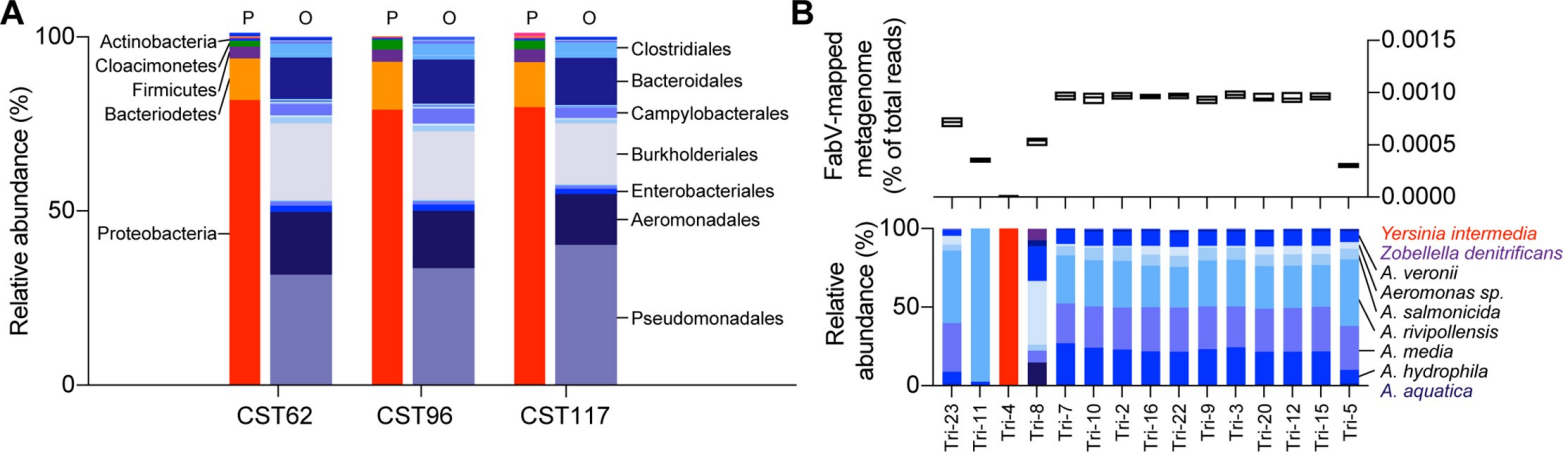
Wastewater community profile and relative abundance and identity of *fabV*-mapped reads. **(A)** Microbiome of WW samples determined by Kraken-based mapping of Illumina HiSeq reads. For each sample, the population composition at the Phylum (P) and Order (O) level is indicated by separate stacked bars. Most frequent taxa are labelled. Complete community profile is available in S1 Table. **(B)** Floating bar graph (center line at mean) showing relative abundance of metagenomic sequence reads mapped to each fabV homolog expressed as a percentage of total reads (upper panel). Species identification of terminal reads mapping to each *fabV* shown as relative abundance (lower panel).

Given the dominance of *Aeromonas*-associated TCS^R^ genes in metagenomic libraries, we characterized the microbial metagenome of the WW DNA samples (CST062, CST096, and CST117). The most abundant phyla (by relative abundance of all mapped reads) were Proteobacteria (80.1 ± 0.82%), Bacteroidetes (12.9 ± 0.52%), and Firmicutes (3.5 ± 0.12%) (Fig 3A; S1 Table). Among Proteobacteria, Pseudomonadales (35.0 ± 2.6%;), Burkholderiales (19.9 ± 1.3%), Aeromonadales (16.4 ± 0.98%), and Campylobacterales (3.4 ± 0.37%) were most abundant. For *Aeromonas*, reads were assigned to *A*. *hydrophila* (5.2 ± 2.18%), *A*. *media* (4.4 ± 0.18%), *A*. *salmonicida* (1.4 ± 0.36%), *A*. *veronii* (0.6 ± 0.06%) and *A*. *schubertii* (0.2 ±0.17%). The most prevalent of the Pseudomonadales was *Acinetobacter* (17.9 ± 1.10%), and for the Burkholderiales it was *Acidovorax* (7.1 ± 0.34%). The relative abundance of reads mapping to *fabV* indicated that the least abundant gene was the *fabV* encoded by Tri-4, whereas *fabV* associated with *Aeromonas* spp. was the most abundant (Fig 3B, upper). To identify species associated with each *fabV*, reads mapping to and extending beyond the terminal ends of *fabV* were filtered and aligned with BLAST. Excepting the Tri-4 *fabV*, terminal reads had the highest identity to *Aeromonas* spp., predominantly *A*. *rivipollensis*, *A*. *media*, and *A*. *salmonicida* (Fig 3B, lower).

### Frequency of triclosan resistance in wastewater and clinical isolates

For *E*. *coli*, the triclosan MIC90 was determined to be 0.125 mg/L. Three *E*. *coli* isolates exhibited up to a 64-fold higher triclosan MICs (2 to 8 mg/L), and were putative ESBL-producers as they were resistant to ampicillin, ceftazidime, and ceftiofur (Table 1). The putative *fabI* gene and promoter from these isolates were cloned into pGEM, but this did not decrease the sensitivity of *E*. *coli* DH5α to triclosan, suggesting that differences in MIC between these *E*. *coli* strains were not due to *fabI* mutations.

**Table 1:** Triclosan MIC distribution (mg/L) for *E*. *coli* and *Enterococcus* spp. derived from wastewater (WW) or from clinical sterile (SS) and non-sterile sites (SS) or vancomycin-resistant *Enterococcus faecium*.

Bacteria	Source	Triclosan MIC distribution (mg/L)															*N*	MIC90
		0.0078	0.0156	0.0313	0.0625	0.125	0.25	0.5	1	2	4	8	16	32	64	128		
*E*. *coli*	WW Putative ESBL^a^	2	3	6	20	14	0	1	0	1	0	2	0	0			49	0.125
*E*. *coli*	WW Non-selected	1	2	9	14	19	3	2	0	0	0	0	0	0			50	0.125
*E*. *faecalis*	WW					0	0	0	0	1	3	7	31	9	5	0	56	32
*E*. *faecalis*	Clinical NS					0	0	1	1	1	5	17	26	19	6	5	81	64
*E*. *faecalis*	Clinical SS					0	0	0	0	0	2	7	13	7	0	1	30	32
*E*. *faecium*	WW					0	0	0	0	0	3	8	21	5	1	0	38	32
*E faecium*	Clinical NS					0	0	0	1	2	3	5	11	5	0	0	27	32
*E*. *faecium*	Clinical SS					0	0	0	0	0	0	4	1	2	1	0	8	64
*E*. *faecium*	Clinical VRE					0	0	0	0	2	15	26	74	27	4	1	149	32

*N* indicates number of strains tested. MIC90 indicates the minimum triclosan concentration inhibiting 90% of isolates tested. Shading indicates triclosan concentrations not tested.

^a^*E*. *coli* isolated from wastewater (WW) with enrichment for putative extended-spectrum β lactamase (ESBL)-producing bacteria.

**Fig 4:**
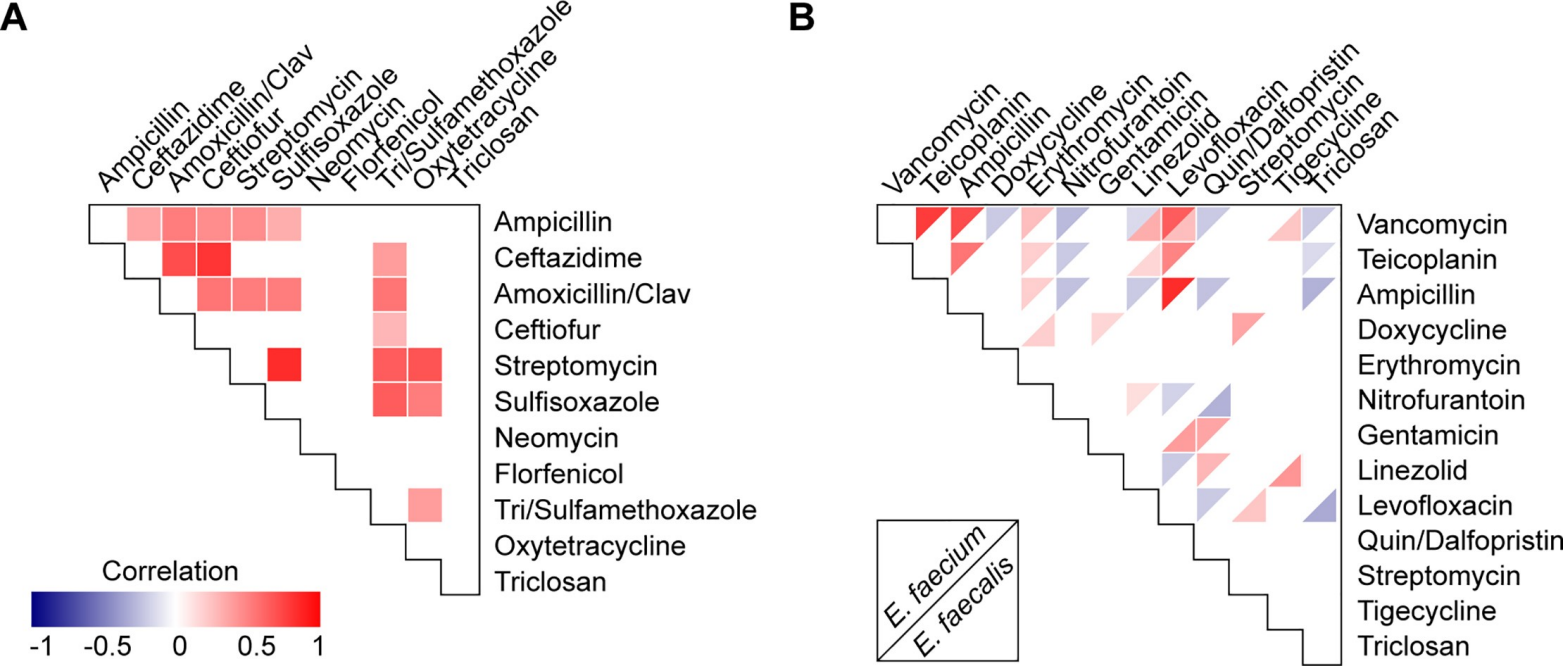
Triclosan MIC correlation with antimicrobial susceptibility profiles for *E*. *coli and Enterococcus* spp. **(A)** Spearman rank-order correlation for *E*. *coli* triclosan MICs and antimicrobial susceptibility profiles for drugs tested. Positive correlations are indicated by correlation strength with red; negative correlations are indicated with blue. **(B)** Spearman rank-order correlation for *E*. *faecium* (upper triangles) and *E*. *faecalis* (lower triangles) triclosan MICs and antimicrobial susceptibility profiles. For both *E*. *coli* and *Enterococcus* spp. correlations, isolates from all sources are grouped. Only significant (P < 0.05) correlations are shown.

The MIC90 for *E*. *faecalis* WW and sterile site (SS) isolates was 32 mg/L, with a modest shift to 64 mg/L for isolates from non-sterile (NS) sites (Table 1). The MIC90 for WW, NS, and VRE *E*. *faecium* was 32 mg/L, and 64 mg/L for SS isolates (Table 1). For *E*. *coli*, even though higher triclosan MIC isolates were ampicillin-resistant, there was no significant correlation between increased triclosan MIC and ampicillin resistance, nor to any other drug (Fig 4A). In contrast, negative correlations (p < 0.05) were found between the triclosan MIC and levofloxacin resistance in *E*. *faecalis*, and between triclosan and vancomycin, teicoplanin, and ampicillin in *E*. *faecium* (Fig 4B).

## Discussion

This study characterized the TCS^R^ gene reservoir in an environment likely impacted by triclosan. Our results highlighted the prevalence of triclosan-refractory ENRs potentially linked to *Aeromonas* spp. These are widespread microbes that are frequently found in both WW and treated drinking water, and are associated with fish, shellfish, and waterfowl [37]. Several species, including *A*. *hydrophila*, *A*. *caviae*, *A*. *media*, *A*. *schubertii*, *A*. *veronii* biovar sobria, and *A*. *veronii* biovar veronii are emerging human pathogens and the causative agents of gastroenteritis, wound and soft tissue infections, necrotizing fasciitis, urinary tract infections, cystic fibrosis, and septicemia [38, 39]. The fish pathogen, *A*. *salmonicida* subsp. *salmonicida* harbors a TCS^R^ FabV ENR homolog with high identity with ENRs in other fish pathogens, including *Edwardsiella* spp., *Vibrio* spp., and *Flavobacterium* spp. [40]. Our functional work suggests that TCS^R^ FabV homologs may be ubiquitous in *Aeromonas* spp. Intrinsic TCS^R^ in the related γ-Proteobacteria *Pseudomonas aeruginosa*, is also due to FabV [10, 17]. Although Pseudomonadales were more prevalent than Aeromonadales in our WW samples, no TCS^R^ determinants were predicted to have originated from Pseudomonadales. This may be because the predominant WW Pseudomonadales were *Acinetobacter*, which typically encode triclosan-sensitive *fabI* in lieu of *fabV*. Similarly, FabI is the terminal ENR in *Acidovorax*, the most abundant *Burkholderiales* in this WW metagenome. Several *fabV* had highly divergent sequences from previously characterized FabV in *V*. *cholerae*, *P*. *aeruginosa*, and *A*. *salmonicida*. With the exception of the *fabV* in cosmid Tri-4, which was identical to that found in some *Yersinia* spp., all but one shared the most identity with putative *fabV* in *Aeromonas* spp. and *Serratia* spp. Although rare, *Y*. *intermedia* and *Y*. *enterocolitica* were present in the WW microbiome (0.12 ± 0.032% of total reads) and only 17 reads (out of ~140M) mapped to *fabV*. Thus, the cosmid library screen functionally selected a rare insert, validating comprehensive functional genomics approaches as powerful methods of identifying new genes or resistance determinants. Despite this, inherent drawbacks of using functional screening of metagenomic libraries for studying TCS^R^ include the fact that they may be unable to reveal the true depth and variety of TCS^R^ (and other AMR) mechanisms, particularly from uncultured organisms whose genes might be incompatible with the library host strain genetics. For example, if TCS^R^ determinants were not expressed or translated, toxic, encoded by a large number of non-contiguous genes, or otherwise irrelevant in the *E*. *coli* host they would not be identified. Additionally, metagenomic libraries frequently exhibit cloning bias compared to the original sample, and are time-consuming and challenging to construct [41].

The functional screening aspect of this work identified *Aeromonas* and *Yersinia* genera as likely origins of TCS^R^ in WW. This is a clear disconnect with the sentinel *Enterococcus* and *E*. *coli* that we collected. Our work suggests that future studies targeted at isolates of *Aeromonas* spp., *Yersinia* spp., and other related species might reveal additional TCS^R^ mechanisms. Although previous work has established triclosan MICs for the bacterial species in our study, clear susceptibility criteria for triclosan do not exist [42, 43]. Triclosan susceptibility criteria or breakpoints are difficult to establish, primarily because triclosan may be formulated into products or used at concentrations much greater than is typical for antimicrobials, often beyond the highest MIC of intrinsically TCS^R^ bacteria. As a result, “resistant” bacteria could be classified as susceptible using standard methodologies. However, many triclosan-containing products are diluted during discharge, and can bioaccumulate in downstream environments [44]. Within waste streams, microbes may be exposed to a wide spectrum of triclosan concentrations [43, 45], such that low-level increases in TCS^R^ could enhance survival. In comparison to MIC90s for *E*. *coli*, *E*. *faecalis*, and *E*. *faecium* in a recent study [42], our MIC90 results are lower for *E*. *coli* (0.125 vs. 0.5 mg/L) and higher for *E*. *faecalis* (32 vs. 8 mg/L) and *E*. *faecium* (32 vs. 8 mg/L). However, this may be due to differences in methodology as we elected to use optical density to estimate bacterial growth as opposed to visual observation, as we found that the low solubility of triclosan interfered with sight-based MIC determination. Furthermore, the MICs we reported are more in-line with previously reported minimal bactericidal concentrations (MBC; the sterilizing drug concentration) and MBC-based epidemiological cut-off values (ECOFF; the drug concentration to which 99.9% of the population is susceptible) [42].

Two putative ESBL-producing *E*. *coli* exhibited triclosan MICs 64-fold higher than the MIC90. The *fabI* genes from these strains did not confer TCS^R^, suggesting the presence of unknown resistance or tolerance mechanisms. These isolates were resistant to ampicillin, ceftazidime, and ceftiofur, and one was also neomycin-resistant. Taken together, it may be useful to routinely select for ESBL-producing bacteria as candidates for biocide-resistance screening, and to assess the impact of triclosan on the dissemination of ESBLs. Paradoxically, most other putative ESBL-producing isolates were frequently MDR (6 drugs, median), but this did not correlate with elevated triclosan MICs. Negative correlations were observed between elevated triclosan MICs and other drugs for both *E*. *faecalis* and *E*. *faecium*. This could indicate fitness costs and selective pressures that trade-off MDR for increased triclosan “resistance”. Triclosan exposure has been associated with increased diversity and abundance of AMR determinants [43]. Thus, it would be interesting to explore triclosan fitness cost/benefits with additional natural isolates with known triclosan exposure histories.

## Supporting information

S1 Table Taxonomic distribution of WW microbiome reads classified by Kraken. (XLSX)
